# Progress and Pollution: Port Cities Prepare for the Panama Canal Expansion

**DOI:** 10.1289/ehp.120-a470

**Published:** 2012-12-03

**Authors:** Andrea Hricko

**Affiliations:** **Andrea Hricko** has written previously for *EHP* on environmental health impacts related to ports. She has developed community–academic partnerships to educate the public and address these issues as part of her work with the NIEHS-funded Southern California Environmental Health Sciences Center, based at the University of Southern California, with additional support from foundations.

Early in the planning of the Panama Canal, Navy Commander Thomas Oliver Selfridge, Jr., wrote that “advantageous as an interoceanic canal would be to the commercial welfare of the whole world, it is doubly so for the necessities of American interests.”^^1^^ And indeed, since the Canal opened in 1914, it has been the main conduit for ocean-going ships carrying trade worldwide. Today the United States ranks number one in tons of cargo passing through the Canal (China ranks number two).^^2^^ In 2011 nearly 13,000 ocean-going cargo ships made the passage.^^3^^

Starting in the late 1950s (and expanding rapidly thereafter), internationally traded goods started being shipped in large metal containers, making it possible to load and unload cargo by machine instead of by hand and spurring the manufacture of larger ships.^^4^^ “Panamax” ships were designed to be just small enough to squeeze through the locks of the Canal.^^5^^ Today, even larger ships—called “post-Panamax” because they are too large to fit through the Canal^^6^^—make up 16% of the world’s container fleet but account for nearly half the fleet’s cargo capacity.^^7^^ To allow these larger ships to transit the Canal and increase its ability to handle higher volumes of ships, Panama is building a third set of locks, with construction expected to be finished in 2015.^^7^^

The Panama Canal expansion has sparked the competitive imagination of East Coast and Gulf Coast (EC/GC) port authorities, who hope to capture some of the 70% of U.S. imports currently controlled by West Coast (WC) ports.^^8^^ Ports typically make their revenues through leases with shipping lines, wharfage fees, and tariffs. So the more containers a port handles, the more money it can make.

Experts at the U.S. Army Corps of Engineers (USACE) call the Panama Canal expansion a likely “game changer” for U.S. trade, potentially redistributing the market share of each coast’s ports, as well as opening up new import and export markets for agricultural and other products along inland waterways.^^7^^ Some have estimated that container volumes at EC/GC ports could more than double from 2012 to 2029.^^7^^ But with this growth come questions about what major initiatives to expand cargo capacity could mean for public health in these port cities.

## Competition for Trade

Whereas most ships transiting the Panama Canal today typically carry 3,200–4,500 TEUs of cargo, shipping experts predict that most ships making the passage after 2015 will be in the 4,500- to 8,000-TEU range,^^9^^ although post-Panamax vessels carrying as many as 12,600 TEUs also will be able to cross.^^10^^ A TEU, or twenty-foot equivalent unit, is a measurement to describe cargo capacity. One TEU represents the capacity of a standard intermodal cargo container measuring 20 × 8 feet.

Many WC ports have 50-foot-deep harbors, ideal for post-Panamax ships. With great fanfare in early 2012, the Port of Long Beach (California) announced it had welcomed to its harbor the *MSC Fabiola*, then the largest container vessel serving the U.S.–Asia trade, with a capacity of 12,500 TEUs^^11^^ (a distinction since claimed by the *MSC Beatrice* at nearly 13,800 TEUs). One shipping newspaper called the arrival of the massive ship a “floating advertisement” for the Port of Long Beach and its deep harbor.^^12^^

When the Panama Canal expansion is complete, the *MSC Fabiola* will be able to pass through the larger canal—but most ports east of the Canal will not be ready to accept it, at least not by 2015. Either their ship channels will not be deep enough to handle the weight of the heavily loaded ship or their bridges will be too low to allow high stacks of containers to pass under them.

In hopes of staying competitive, many EC/GC port authorities (as well as railroads and state highway departments) are taking action to dredge deeper harbors and improve bridges, tunnels, rail lines, and highways to accommodate larger ships and higher cargo volumes. Behind the efforts are implicit hopes—and optimistic forecasts—that the economy will improve and that Asian and other imports will soar in the future.

As one example, the Port Authority of New York and New Jersey is proposing to raise the height of the roadway across the Bayonne Bridge at a cost of $1 billion.^^13^^ The Port of Jacksonville, in Florida, is working with the USACE to examine the benefits and costs of deepening its ship channel from the existing depth of 40 feet to a depth of 50 feet,^^14^^ and Georgia’s Port of Savannah has gotten the go-ahead to dredge its channel, which will cost more than $650 million.^^15^^

Maryland’s Port of Baltimore has a deep harbor, but its old railroad tunnel exiting the port terminals is not tall enough for today’s double-stacked trains to pass through.^^16^^ As a solution, the major railroad company CSX is planning to build a new intermodal rail transfer facility that will facilitate moving double-stacked trains away from the port.^^17^^ The Port of Miami, in Florida, has starting boring twin tunnels that would allow big-rig trucks entering or leaving the port to bypass downtown Miami streets, at a cost of $607 million.^^18^^

Ports have also partnered with railroad companies to build new rail corridors to move containers inland more quickly from EC/GC ports. As one example, the railroad company Norfolk Southern has built the Heartland Corridor, which makes more rail tracks available, allows the loading of double-stacked containers on its trains, and increases freight rail capacity between Virginian ports and the Midwest.^^19^^ Norfolk Southern had to blast through more than two dozen Appalachian Mountain passes in West Virginia, Virginia, and Kentucky so these double-stacked trains could pass through with a higher vertical clearance.^^20^^

## Potential Environmental Health Impacts

To evaluate environmental impacts of these infrastructural enhancements, one must first look to emissions at the ports, starting with the ships themselves. Ships burn bunker fuel—a thick, high-sulfur by-product of traditional fuel-oil refining—and are large contributors to air pollution throughout the world, and especially in port communities.^^21^^ James Corbett, a professor at the University of Delaware School of Marine Science and Policy, has calculated that ship emissions may cause as many as 60,000 deaths a year worldwide from heart disease and cancer.^^22^^

Corbett says the mandatory use of lower-sulfur fuel in ships resulting from implementation of the North American Emission Control Area (ECA)—which went into effect 1 August 2012—has the potential to reduce sulfur dioxide emissions and associated health effects near coastlines. The North American ECA was negotiated between the U.S. Environmental Protection Agency and the International Maritime Organization and requires that ocean-going ships switch to lower-sulfur fuel within 200 nautical miles of the U.S. shoreline.^^23^^ Corbett notes, however, that growth in trade volume could erase the health value of the ECA within one or two decades.

Once ships reach their destinations, ship emissions remain a problem in port harbors. Crews need air conditioning, refrigeration, and other services over the days it takes to unload and reload a ship. Unless the ships are able to plug into electricity, a process referred to as “shore power” or “shoreside power,” they must run their engines to provide these services. Because many port communities already have poor air quality^^24^^ and are often disproportionately lower-income,^^25^^ emissions from ships in harbor can add significantly to local air pollution and to health inequities. (It should be noted that switching to electricity for power while ships are in harbor, instead of burning fossil fuel in auxiliary engines, potentially translates into additional pollution burdens for residents in other locations near the power plants that supply electricity to the port—a topic that is beyond the scope of this article.)

Of particular concern for expanding ports is the increased truck traffic that will result from larger ports, bigger ships, and a higher volume of containers. After leaving a port, each container has to be transferred to a diesel-fueled truck or a train powered by up to four diesel-fueled locomotives to move the cargo to inland destinations. According to the USACE, trucks consume nearly three-quarters of the freight transport fuel used in goods movement, largely a result of fuel inefficiency, and efforts to reduce truck traffic in favor of trains often fail because trucks serve double duty as delivery vehicles.^^7^^

The U.S. Environmental Protection Agency last evaluated diesel particulate matter in 2003 and concluded it is a “likely human carcinogen.”^^26^^ However, in 2012 the International Agency for Research on Cancer (IARC) elevated its own classification of diesel engine exhaust from “probably carcinogenic” to “carcinogenic to humans” on the basis of sufficient evidence that exposure is associated with an increased risk for lung cancer.^^27^^ In California, the state Air Resources Board has estimated that, each year, emissions of fine particulate matter from freight transport activities contribute to 3,700 premature deaths in the state.^^28^^

Corbett believes “expansion of the Panama Canal is not necessarily a win–win–win situation,” and he raises concerns that exhaust from an increase in truck traffic not just in port cities but also along major eastern corridors such as I-95 will need to be addressed. Concerns about traffic congestion from freight transportation led to the formation years ago of an I-95 Corridor Coalition of transportation agencies and officials, with members from Maine to Florida. The coalition today has committees investigating solutions to truck traffic congestion, even looking at shipping between EC ports to avoid additional trucks on I-95.^^29^^

In a forthcoming paper Corbett and colleagues analyze various scenarios on what might happen to emissions of greenhouse gases and other pollutants if significant amounts of WC imports are shifted to EC/GC ports.^^30^^ They consider cargo travel from WC ports to other parts of the country via truck and train, as well as via larger ships going through the Canal to the Atlantic. They conclude there are some reductions in carbon dioxide emissions on a per-container basis for larger ships going through the Canal. But the reductions were nearly negated by the longer distances the ships had to travel.

Rail yard facilities are another source of significant air pollution for port communities, and these facilities also are often located in lower-income and minority communities.^^31^^ The first health impact assessment^^32^^ to be conducted of a U.S. rail yard facility has been funded by a collaboration of foundations looking at a new site selected in Baltimore where cargo will be switched between trains and trucks. Health impact assessments are tools that assist policy makers in better understanding potential health impacts of proposed infrastructure projects or policies.^^33^^ Concerns to be investigated at the Baltimore site include air and noise pollution and substantial increases in truck and locomotive traffic, according to Rebecca Morley, executive director of the National Center for Healthy Housing, which will conduct the assessment.

## A Potential Model for Growth

The side-by-side Ports of Los Angeles and Long Beach offer a potential model for developing programs to reduce diesel exhaust emissions. Those ports’ Clean Air Action Plan (CAAP)^^34^^ has resulted in a significant reduction in diesel emissions since it was adopted in 2006.^^35^^^^,^^^^36^^

The CAAP includes several components: 1) a Clean Trucks Program to phase out older diesel trucks from the ports within five years and replace them with a new generation of clean or retrofitted vehicles; 2) recommendations to eliminate emissions of ultrafine particulate matter; 3) a technology advancement program to reduce emissions from other equipment, including commitments to develop shore power for ships; and 4) a public participation process with environmental organizations and the business community.

The CAAP was adopted with a pledge to reduce existing levels of air pollution by least 45% within five years.^^34^^ As of 1 January 2012 the CAAP bans trucks older than model year 2007 from picking up or dropping off containers at either port.^^37^^ In summer 2012 officials with the Port of Los Angeles issued its annual emissions inventory, which showed the port is in line to meet and in some cases exceed even stricter emission reduction goals for diesel particulate matter and nitrogen oxides by 2014.^^38^^ Meanwhile, the State of California also adopted new air quality regulations to reduce diesel particulate matter in exhaust, requiring that all heavy-duty diesel trucks in the state be equipped with particulate filters by 1 January 2012.^^39^^

Some of the EC/GC ports are far behind Los Angeles and Long Beach in developing environmental programs. But Geraldine Knatz, executive director of the Port of Los Angeles, suggests caution in comparing EC/GC and WC ports, noting that some ports do not have as serious air pollution problems as Southern California and so might not require measures as stringent as those at the California ports. She adds that other ports may not find it feasible to install expensive mitigation measures to reduce pollution, such as electrical shore power for ships. Based on her own experience, however, she recognizes there is a lot more some ports could be doing to address pollution.

Several EC/GC ports now have their own versions of a Clean Truck Program or programs to reduce emissions from other equipment used at the marine terminals. These programs are often voluntary, whereas clean trucks are mandatory at the Ports of Los Angeles and Long Beach. For example, the Port of Houston, in Texas, has a loan program for drivers in hopes of replacing 230 of its oldest trucks. Nearly 40% of the 3,000 drayage trucks serving that port are more than 15 years old.^^40^^ By July 2012, 90 trucks had been replaced.^^40^^ The Port of New York and New Jersey also has a Clean Trucks Program that currently bans trucks older than 1994, but its ban on trucks older than 2007 did not go into effect until 2012,^^41^^ five years behind Los Angeles and Long Beach.

Many ports have begun to replace diesel-powered cranes with less-polluting electric cranes. The Georgia Ports Authority reports that 21 of its 23 container cranes are now powered by electricity;^^42^^ the Port of New York and New Jersey has bought electric cranes, as have the Port of Baltimore^^43^^ and others. Apparently no EC/GC ports currently have shore power installed at their marine terminals for container ships, although the Brooklyn Cruise Terminal, part of the Port of New York and New Jersey, is developing this capability for cruise ships.^^44^^ In addition, at a dock in Baltimore, the Moran Towing Corporation has built shore power plug-ins for tugboats.^^45^^ The Port of Charleston has to date resisted pleas from environmental groups^^46^^ and from the state’s medical society^^47^^ to have its cruise ships plug in to electricity.

There are many more types of potential pollution reduction strategies for ports, rail yards and highways than can be mentioned in this article.^^48^^ One relatively easy solution is to have ships voluntarily reduce their speeds as they come into harbor. Investigators recently demonstrated the benefit of this strategy when they measured the emissions of one Panamax and one post-Panamax ship as they came into the Ports of Los Angeles and Long Beach—when the ships slowed their speeds, emissions of carbon dioxide, nitrogen oxides, and particulate matter dropped significantly.^^49^^ The Southern California ports offer financial incentives to shipping lines to reduce speeds.

## Public Involvement

Public transparency and public involvement opportunities also differ dramatically between many EC/GC ports and Los Angeles and Long Beach. Whereas both the Los Angeles and Long Beach ports videotape their harbor commission meetings and post them online for ease of use by the public, some EC/GC ports, such as the South Carolina Ports Authority, do not post even their board minutes online.^^50^^ The Port of Jacksonville requires a written request to view any meeting minutes.^^51^^

The presence of community-based and environmental justice groups advocating for reducing port-, rail-, and truck-related health impacts is also less apparent in the EC/GC port cities than in Southern California. In 2011 there were more than 15 active environmental and community-based organizations and coalitions in Southern California alone focusing staff time on these impacts,^^52^^ whereas some major EC/GC port cities (e.g., the Port of Virginia, with multiple terminals) have few if any groups monitoring expansion or demanding emission reductions to protect public health.

However, other port communities—including Gulfport, Houston, Charleston, and New York/New Jersey—do have organizations actively advocating for public health considerations in their ports’ expansions.^^52^^ Several lawsuits by environmental and conservation groups have raised questions about increased air pollution due to expansion of port terminals (for example, in Charleston^^53^^) and environmental impacts of dredging (for example, in Savannah, Georgia^^54^^). And at least one group has used the IARC carcinogenicity ruling for diesel particulate matter to support its request for a rigorous environmental impact assessment—in this case, by the U.S. Coast Guard before raising the Bayonne Bridge.^^55^^ In comments submitted to the Board of Commissioners of the Port Authority of New York and New Jersey in August 2012, Cynthia Mellon, community and environmental justice organizer for Ironbound Community Organization in Newark, said, “Our community wants growth and the jobs it will generate, but not at the cost of our health.”^^55^^

Meanwhile, as this story went to press, damages from Hurricane Sandy were still being assessed, and it’s unclear how they will affect EC port authorities’ plans for expansion. All terminals of the Port of New York and New Jersey were shut with no power for nearly a full week after Sandy’s storm surge hit on 29 October 2012. Before that week was up, some cargo ships chose to leave the New York area and headed south to the Port of Virginia to offload their goods.^^56^^

## References

[1] McCullough D. The Path Between the Seas: The Creation of the Panama Canal, 1870–1914. New York, NY:Simon and Schuster (1977).

[2] DOT. Maritime Trade & Transportation, 2007. Washington, DC:Bureau of Transportation Statistics, U.S. Department of Transportation (2008). Available: http://www.bts.gov/publications/maritime_trade_and_transportation/2007/pdf/entire.pdf [accessed 20 Nov 2012].

[3] Panama Canal Authority. Panama Canal Transit Statistics, Fiscal Years 2010 through 2012. Table 1. Miami, FL:Panama Canal Authority (2012). Available: http://www.pancanal.com/eng/op/transit-stats/2012-Table01.pdf [accessed 20 Nov 2012].

[4] World Shipping Council. History of Containerization [website]. Washington, DC:World Shipping Council (2012). Available: http://www.worldshipping.org/about-the-industry/history-of-containerization [accessed 20 Nov 2012].

[5] Knight K. IWR White Paper. The Implications of Panama Canal Expansion to U.S. Ports and Coastal Navigation Economic Analysis. Washington, DC:U.S. Army Corps of Engineers (Dec 2008). Available: http://www.iwr.usace.army.mil/docs/iwrreports/WhitePaperPanamaCanal.pdf [accessed 20 Nov 2012].

[6] Tirschwell P. Not ready for post-Panamax ships by 2014? Relax. J Commerce (26 Oct 2011). Available: http://goo.gl/GbqIz [accessed 20 Nov 2012).

[7] USACE. U.S. Port and Inland Waterways Modernization: Preparing for Post-Panamax Vessels. Washington, DC:U.S. Army Corps of Engineers (20 Jun 2012). Available: http://goo.gl/KSl1D [accessed 20 Nov 2012].

[8] Mongelluzzo B. New locks could benefit both coasts, Aleman says. J Commerce (19 Sep 2009). Available: http://goo.gl/vMdEM [accessed 20 Nov 2012].

[9] Armbruster B. The port community game changer: expansion of the Panama Canal will reshape global trade patterns. Cargo Business News (10 Feb 2012). Available: http://www.cargobusinessnews.com/Feb10/portcomm_game_changer.html [accessed 20 Nov 2012].

[10] Sabonge R. Expansion of the Panama Canal: Potential Impact on Asia—East Coast/Golf [sic] Trade. Miami, FL:Panama Canal Authority (21 Oct 2009). Available: http://goo.gl/VtV6S [accessed 20 Nov 2012].

[11] Port of Long Beach. Port Welcomes Largest Container Ship [press release]. Long Beach, CA:Port of Long Beach (16 Mar 2012). Available: http://www.polb.com/news/displaynews.asp?NewsID=979&TargetID=1 [accessed 20 Nov 2012].

[12] Virginia and Baltimore ports winning the race to 50 foot depth; East Coast ports behind on accommodating bigger ships. Southeast Shipping News (23 Mar 2012). Available: http://goo.gl/2LcOL [accessed 20 Nov 2012].

[13] Bayonne Bridge Navigational Clearance Program [website]. Jersey City, NJ:Port Authority of New York and New Jersey (2012). Available: http://www.panynj.gov/bayonnebridge [accessed 20 Nov 2012].

[14] Harbor Deepening [website]. Jacksonville, FL:Jacksonville Port Authority. Available: http://www.jaxport.com/cargo/maritime-resources/harbor-deepening [accessed 20 Nov 2012].

[15] Savannah harbor expansion project gets the go-ahead. Marine Log (14 Nov 2012). Available: http://goo.gl/fSNQA [accessed 20 Nov 2012].

[16] Halsey III, A. Aging Baltimore tunnel a threat to shipping economy for the city and Maryland. Washington Post, Traffic section, online edition (28 Mar 2012). Available: http://goo.gl/KH5G1 [accessed 20 Nov 2012].

[17] Dresser M. City train yard tentatively selected as site of port transfer station. The Baltimore Sun, Collections section, CSX Transportation subsection, online edition (11 Sep 2012). Available: http://goo.gl/cuKk2 [accessed 20 Nov 2012].

[18] Port of Miami Tunnel, FAQs. Financial [website]. Miami, FL:Florida Department of Transportation. Available: http://www.portofmiamitunnel.com/faqs/financial/ [accessed 20 Nov 2012].

[19] Dubin CH. Paving the path to progress. Inbound Logistics (Jan 2010). Available: www.inboundlogistics.com/cms/article/paving-the-path-to-progress/ [accessed 20 Nov 2012].

[20] Norfolk Southern Corporation. The Heartland Corridor has arrived. BizNS 2(6)1–5 (Nov/Dec 2010). Norfolk, VA:Norfolk Southern Corporation. Available: http://www.nscorp.com/nscorphtml/bizns/bzns1210/BizNSNovDec10_Web.pdf [accessed 20 Nov 2012].

[21] McCarthy JE. Air Pollution and Greenhouse Gas Emissions from Ships. Washington, DC:Congressional Research Service (23 Dec 2009). Available: http://crs.ncseonline.org/nle/crsreports/10Jan/RL34548.pdf [accessed 20 Nov 2012].

[22] Corbett JJ Mortality from ship emissions: a global assessment. Environ Sci Technol 41 24 8512 8518; http://dx.doi.org/. 10.1021/es071686z 18200887

[23] EPA. Designation of North American Emission Control Area to Reduce Emissions from Ships. Washington, DC:Office of Transportation and Air Quality, U.S. Environmental Protection Agency (Mar 2010). Available: http://www.epa.gov/otaq/regs/nonroad/marine/ci/420f10015.pdf [accessed 20 Nov 2012].

[24] Port Communities in Non-Attainment Areas for National Ambient Air Quality Standards [website]. Alexandria, VA:American Association of Port Authorities (2012). Available http://www.aapa-ports.org/Issues/content.cfm?ItemNumber=1278 [accessed 20 Nov 2012].

[25] Rosenbaum A Analysis of diesel particulate matter health risk disparities in selected US harbor areas. Am J Public Health 101 S1 S217 S223 2011); http://dx.doi.org/. 10.2105/AJPH.2011.300190 21836118 PMC3222501

[26] EPA. Diesel Engine Exhaust Quickview (CASRN NA) [website]. Washington, DC:Integrated Risk Information System (IRIS), U.S. Environmental Protection Agency (updated 20 Nov 2012). Available: http://goo.gl/ndh13 [accessed 20 Nov 2012].

[27] Benbrahim-Tallaa L. Carcinogenicity of diesel-engine and gasoline-engine exhausts and some nitroarenes. Lancet Oncol 13 7)663 664 2012); http://dx.doi.org/. 10.1016/S1470-2045(12)70280-2 22946126

[28] ARB. Draft Staff Report. Methodology for Estimating Premature Deaths Associated with Long-term Exposures to Fine Airborne Particulate Matter in California. Sacramento, CA:California Air Resources Board (ARB), California Environmental Protection Agency (7 Dec 2009). Available: http://www.arb.ca.gov/research/health/pm-mort/pm-mortdraft.pdf [accessed 20 Nov 2012].

[29] I-95 Corridor Coalition. A 2040 Vision for the I-95 Coalition Corridor: Supporting Economic Growth in a Carbon-Constrained Environment. Rockville, MD:I-95 Corridor Coalition (2008). Available: http://goo.gl/mZKwX [accessed 20 Nov 2012].

[30] Corbett JJ Panama Canal expansion: emission changes from possible US west coast modal shift. Carbon Manag [in press].

[31] ICF International. Report to USEPA on the Locomotive and Marine Engine Rule. U.S. EPA Docket EPA-HQ-OAR-2003. Appendix H. Age, Income, and Racial/Ethnic Composition of Populations Exposed to DPM in the Vicinity of Rail Yards and Terminals. Available: http://hydra.usc.edu/scehsc/web/resources/Map/Railyards/EPA-HQ-OAR-2003-0190-0752.8.pdf [accessed 20 Nov 2012].

[32] Wernham A. Health impact assessments are needed in decision making about environmental and land-use policy. Health Aff 30 5 947 956 2011); http://dx.doi.org/. 10.1377/hlthaff.2011.0050 21555479

[33] Health Impact Project [website]. Washington, DC:Health Impact Project, The Pew Charitable Trusts (2012). Available: http://www.healthimpactproject.org/hia [accessed 20 Nov 2012].

[34] San Pedro Bay Ports Clean Air Action Plan [website]. San Pedro, CA:Port of Los Angeles, City of Los Angeles (2012). Available: http://www.portoflosangeles.org/environment/caap.asp [accessed 20 Nov 2012].

[35] Port of Los Angeles. Emissions Inventory Highlights—2011. San Pedro, CA:Port of Los Angeles (Jul 2012). Available: http://www.portoflosangeles.org/pdf/2011_Air_Emissions_Inventory_Highlights.pdf [accessed 20 Nov 2012].

[36] Port of Long Beach. Emission Inventory Documents [website]. Long Beach, CA:Port of Long Beach (2012). Available: http://www.polb.com/environment/air/emissions.asp [accessed 20 Nov 2012].

[37] About the Port of Los Angeles Clean Truck Program. San Pedro, CA:Port of Los Angeles, City of Los Angeles (2012). Available: http://www.portoflosangeles.org/ctp/idx_ctp.asp [accessed 20 Nov 2012].

[38] Port of Los Angeles. More Cargo, Cleaner Air at the Port of Los Angeles [press release]. San Pedro, CA:Port of Los Angeles (2 Aug 2012). Available: http://www.portoflosangeles.org/newsroom/2012_releases/news_080212_Inventory_Air_Emissions.asp [accessed 20 Nov 2012].

[39] ARB. On-Road Heavy-Duty Diesel Vehicles (In-Use) Regulation [website]. Sacramento, CA:Air Resources Board, California Environmental Protection Agency (2012). Available: http://www.arb.ca.gov/msprog/onrdiesel/onrdiesel.htm [accessed 20 Nov 2012].

[40] EDF. Cleaner Air in Port Cities, Thanks to New Trucks. New York, NY:Environmental Defense Fund (5 Oct 2012). Available: http://www.edf.org/health/cleaner-air-port-cities-thanks-new-trucks [accessed 20 Nov 2012].

[41] Port Authority of New York and New Jersey. Port Authority Launches Program to Replace Older, More Polluting Trucks Serving the Port of NY/NJ [press release]. New York, NY:Port Authority of New York and New Jersey (10 Mar 2010). Available: http://www.panynj.gov/press-room/press-item.cfm?headLine_id=1267 [accessed 20 Nov 2012].

[42] Leach PT. Savannah to switch to electric-powered cranes. J Commerce (27 Jan 2012). Available: http://www.joc.com/port-news/savannah-switch-electric-powered-cranes_20120127.html [accessed 20 Nov 2012].

[43] Maryland Port Administration. New Supersized Container Cranes for 50-Foot Berth Nearing Arrival at Port of Baltimore [press release]. Baltimore, MD:Maryland Port Administration (30 May 2012). Available: http://mpa.maryland.gov/_media/client/News-Publications/2012/press/053012press.pdf [accessed 20 Nov 2012].

[44] Port Authority of New York and New Jersey. Port Authority to Proceed with Installation of Shore Power Technology at Brooklyn Cruise Terminal. New York, NY:Port Authority of New York and New Jersey (28 Jun 2012). Available: http://www.panynj.gov/press-room/press-item.cfm?headLine_id=1604 [accessed 20 Nov 2012].

[45] Jackson NM. Moran pulls its weight with environmental efforts. Port of Baltimore Magazine, Mar/Apr 2011 (30 Mar 2011). Available: http://pobdirectory.com/news/2011/03/30/marchapril-2011-port-of-baltimore-magazine/ [accessed 20 Nov 2012].

[46] Smith B. Historic Charleston fights over docking of cruise ships. USA Today, Travel section, online edition (22 Apr 2012). Available: http://goo.gl/wktc9 [accessed 20 Nov 2012].

[47] State medical association advocates for shoreside power for cruise ships (editorial). The Post and Courier (Charleston SC), Editorial section, online edition (6 May 2012). Available: http://www.postandcourier.com/article/20120506/PC1002/120509447 [accessed 20 Nov 2012].

[48] Cannon JS. U.S. Container Ports and Air Pollution: A Perfect Storm. An Energy Futures, Inc. Study (2008). Boulder, CO:Energy Futures, Inc. (2008). Available: http://s3.amazonaws.com/energy-futures.com/port_study_ef.pdf [accessed 20 Nov 2012].

[49] Khan MY Greenhouse gas and criteria emission benefits through reduction of vessel speed at sea. Environ Sci Technol 46 22 12600 12607 2012); http://dx.doi.org/. 10.1021/es302371f 22974075

[50] Freedom of Information Act, South Carolina State Ports Authority [website]. Available: http://www.port-of-charleston.com/About/statistics/FOIA.asp [accessed 20 Nov 2012].

[51] Public Records [website]. Jacksonville, FL:Jacksonville Port Authority (JAXPORT). Available: http://www.jaxport.com/about-jaxport/public-records [accessed 20 Nov 2012].

[52] Matsuoka M, et al. Global Trade Impacts: Addressing the Health, Social and Environmental Consequences of Moving International Freight through Our Communities. Los Angeles, CA:Oxy Scholar, Occidental College (Mar 2011). Available: http://scholar.oxy.edu/uep_faculty/411/ [accessed 20 Nov 2012].

[53] Bird A. Port, conservation league settle lawsuit over new port terminal. The Post and Courier, News section, online edition (updated 19 Mar 2012). Available: http://www.postandcourier.com/article/20100806/PC16/308069969 [accessed 20 Nov 2012].

[54] Bynum R. Army Corps of Engineers gets final approval for $652 million deepening of Savannah Harbor. Associated Press, via The Augusta Chronicle, online edition (27 Oct 2012). Available: http://goo.gl/eIm4y [accessed 20 Nov 2012].

[55] Mellon C. Statement by Cynthia Mellon, Community and Environmental Justice Organizer, Ironbound Community Corporation, Coalition for Healthy Ports, New Jersey Environmental Justice Alliance. Presented to the Board of Commissioners of the Port Authority of New York and New Jersey. 1 Aug 2012. Available: http://www.cleanwateraction.org/positionstatement/pressstatements [accessed 20 Nov 2012].

[56] Morris B, McWhirter C. Cargo ships seek alternate ports. Wall Street Journal, Business section, online edition (updated 2 Nov 2012). Available: http://goo.gl/ZUhRC [accessed 20 Nov 2012].

